# Transcriptome Analysis and Identification of a Female-Specific SSR Marker in *Pistacia chinensis* Based on Illumina Paired-End RNA Sequencing

**DOI:** 10.3390/genes13061024

**Published:** 2022-06-07

**Authors:** Xiaomao Cheng, Fei Wang, Wen Luo, Jingge Kuang, Xiaoxia Huang

**Affiliations:** Southwest Landscape Architecture Engineering Research Center of State Forestry and Grassland Administration, College of Landscape Architecture and Horticulture Sciences, Southwest Forestry University, Kunming 650224, China; xmcheng0103@gmail.com (X.C.); wangfei_icon@163.com (F.W.); luo2022050516@163.com (W.L.); kjg1106@163.com (J.K.)

**Keywords:** *Pistacia chinensis*, transcriptome, EST-SSR, sex identification

## Abstract

*Pistacia chinensis* Bunge (*P. chinensis*), a dioecious plant species, has been widely found in China. The female *P. chinensis* plants are more important than male plants in agricultural production, as their seeds can serve as an ideal feedstock for biodiesel. However, the sex of *P. chinensis* plants is hard to distinguish during the seedling stage due to the scarcity of available transcriptomic and genomic information. In this work, Illumina paired-end RNA sequencing assay was conducted to unravel the transcriptomic profiles of female and male *P. chinensis* flower buds. In total, 50,925,088 and 51,470,578 clean reads were obtained from the female and male cDNA libraries, respectively. After quality checks and de novo assembly, a total of 83,370 unigenes with a mean length of 1.3 kb were screened. Overall, 64,539 unigenes (77.48%) could be matched in at least one of the *NR*, *NT*, *Swiss-Prot*, *COG*, *KEGG*, and *GO* databases, 71 of which were putatively related to the floral development of *P. chinensis.* Additionally, 21,662 simple sequence repeat (SSR) motifs were identified in 17,028 unigenes of *P. chinensis*, and the mononucleotide motif was the most dominant type of repeats (52.59%) in *P. chinensis*, followed by dinucleotide (22.29%), trinucleotide (20.15%). The most abundant repeats were AG/CT (13.97%), followed by AAC/GTT (6.75%) and AT/TA (6.10%). Based on these SSR, 983 EST-SSR primers were designed, 151 of which were randomly chosen for validation. Of these validated EST-SSR markers, 25 SSR markers were found to be polymorphic between male and female plants. One SSR marker, namelyPCSSR55, displayed excellent specificity in female plants, which could clearly distinguish between male and female *P. chinensis*. Altogether, our findings not only reveal that the EST-SSR marker is extremely effective in distinguishing between male and female *P. chinensis* but also provide a solid framework for sex determination of plant seedlings.

## 1. Introduction

At present, the divergence in the relationship between fossil energy consumption and global oil demand is becoming increasingly prominent. The volatility of international crude oil market and the environmental issues caused by the wide-scale use of fossil fuels have become two major areas of focus [1]. Moreover, it is bound to develop diversified, re-biochemical, and clean energy for humans. Therefore, the development of new biomass energy is one of the most important directions to solve the global energy crisis. *P. chinensis* is a dioecious plant species with wind-pollinated apetalous flowers, and its biomass can serve as a potentially important renewable energy option [2]. This species is commonly found in China and has many distinctive features. For instance, it can tolerate dry conditions and grow in alkaline or acidic soil [3]. The oil content of *P. chinensis* seed is typically higher than 40%, which is a non-drying type. The sixteen alkyl value of biodiesel produced by *P. chinensis* is generally up to 51.3 [4]. Given its broad distribution and high yields, *P. chinensis* has become a possible new source for biodiesel production.

*P. chinensis* is a long-lived species with a lengthy period of juvenility (typically >8 years). To gain the optimum economic benefits from large-scale production of *P. chinensis*, it is necessary to manipulate the ratio of females to males in the early stages. Thus far, the specific marker for the sex identification of *P. chinensis* is still lacking, particularly at the seedling phase. Over the past decades, few genes or gene sequences have been discovered in *Pistacia* species, which can be employed as the sex-linked markers [5,6,7,8,9,10]. A random amplified polymorphic DNA (RAPD) marker OPO08945 has been identified as a single sex marker for differentiating female and male *P. vera* seedlings but not for other *Pistacia* species [5]. Sequence-characterized amplified region (SCAR) marker is one of the stable molecular markers derived from RAPD. A female-specific SCAR marker has been detected in *P. vera* by touchdown PCR program [6], and this SCAR marker can be used to effectively distinguish gender in wild *Pistacia* species, including *P. atlantica* and *P. khinjuk*. In *P. vera*, theSCAR markers demonstrated false-negative and false-positive results in females and males, respectively, in a segregating F_1_ population (“Siirt” × “Bağyolu”) as well as some germplasm [8]. However, the study reported that the identified SCAR marker was not effective to distinguish sex in other wild *Pistacia* species [8]. To develop sex-linked markers for the marker-assisted selection (MAS) in breeding programs, eight loci from seven RAD reads were successfully able to distinguish the sexes in *P. vera* [8,9], and a female heterogamete ZW/ZZ sex-determination system was first reported [8]. In *P. chinensis*, two sex-specific primers were identified and amplified PCR fragments of 1242 and 473 bp among female plants, but only one fragment was successfully converted to a SCAR marker. Besides, a 636 bp DNA fragment was detected in all six female samples but not in all the six male samples, and the maker was not verified in a large population, so it is very difficult to determine the effectiveness of the marker [10]. Despite that, MAS for identifying the gender of *P. chinensis* seedlings is still lagging behind other *Pistacia* species, including *P. atlantica*, *P. khinjuk,* and *P. vera* [8]. Therefore, a cost-effective and rapid molecular method is needed to detect the sex-specific markers in *P. chinensis*.

RNA sequencing (RNA-seq) is a high-throughput, next-generation technology that possesses several distinct advantages in analyzing the fine structure of a transcriptome [11]. Apart from genome sequencing, a massive amount of sequence information can be obtained from transcriptome sequencing [12]. RNA-Seq is an important tool for transcriptomic analysis, gene identification, and molecular marker discovery in different organisms [13,14,15]. And RNA-seq is also used for identification of sex-related processes [16,17,18,19,20]. The sex-linked markers of *Pistacia* genus have been mostly investigated by inter simple sequence repeat (ISSR), RAPD, and SCAR [5,6,7,8,9,10]. However, there are no simple sequence repeat (SSR) markers reported on *P. chinensis*. SSR markers are far more common than other molecular markers due to their high genomic coverage, codominant inheritance, hyper variable sequences, high effectiveness, and excellent reliability [21].

In this research, the transcriptomic analysis of *P. chinensis* was carried out using Illumina HiSeq 2000 platform. We sampled the pooled transcriptomes of flower buds of *P. chinensis*, constructed a large-scale expressed sequence tag (EST) database, and developed a gender-specific SSR marker. The sex-linked SSR markers were further verified in a large population of *P. chinensis* plants. To our knowledge, few studies focus on SSR marker discovery in *P. chinensis* based on RNA-seq application [22].

## 2. Materials and Methods

### 2.1. Plant Sampling and RNA Extraction

**Figure 1:**
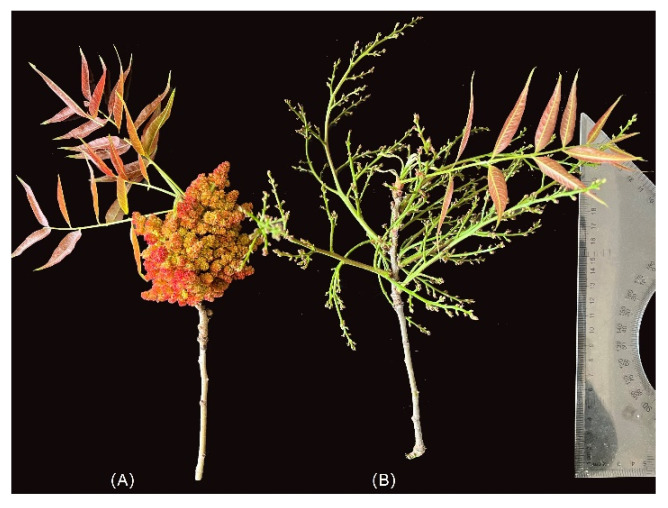
Male (**A**) and female (**B**) inflorescences of *P. chinensis*.

The flowers of *P. chinensis* are unisexual and clustered in axillary panicles. Flowers are small, and perianth segments are lanceolate or narrowly lanceolate with a length of about 1.5–2 mm and with a pedicel of about 1 mm. Bracts are lanceolate or narrowly lanceolate, with a length of about 1.5–2 mm. Male inflorescences are closely arranged with a length of 6–7 cm, and the female inflorescences are loosely arranged with a length of 15–20 cm (Figure 1). The flower buds of *P. chinensis* (more than 100 buds per plant) were pooled from 3 female trees and 3 male trees (at least 10 m tall), respectively, in Kunming, Yunnan, southwest of China. All samples were snap-frozen in liquid nitrogen and kept at –80 °C until further analysis. Total RNA was extracted with the EASY spin Plus Plant RNA Kit following the manufacturer’s instructions (Aidlab Biotech, Beijing, China). The purity and quantity of RNA samples were determined by a NanoDrop 2000 spectrophotometer (Life Technologies, CA, USA). Only those samples with an A260/A280 of >1.8 and an A260/A230 ratio of >1.8 were selected for subsequent analysis. Final quality assessment was performed using the Bioanalyzer RNA 6000 Nano assay (Agilent Technologies, Santa Clara, CA, USA) prior to deep sequencing. The qualified samples with RNA integrity number (RIN) of >6.5 were selected for further analysis. DNase I (RNase-free) treatment was performed on all RNA samples in order to eliminate possible DNA contamination.

### 2.2. cDNA Library Construction and Paired-End Sequencing

cDNA libraries were constructed with the pooled RNA samples (5 μg) by using the NEB Next Ultra RNA Library Prep Kit for Illumina (NEB, Ipswich, MA, USA) according to the manufacturer’s instructions. The quality and quantity of cDNA libraries were assessed using the StepOnePlus Real-Time PCR System (Applied Biosystems, Foster City, CA, USA) and Agilent 2100 Bioanaylzer (Agilent Technologies, Santa Clara, CA, USA). Two cDNA libraries of female and male *P. chinensis* plants were subjected to paired-end sequencing on an Illumina HiSeq 4000 platform (1Gene Company, Hangzhou, China).

### 2.3. Data Pre-Processing and De Novo Assembly

To obtain high-quality clean data, the raw reads were filtered using in-house Perl scripts. The Q20, Q30, GC content, and sequence duplication level of the clean sequences were determined. De novo assembly of the transcriptomic data was conducted with a Trinity assembler (trinityrnaseq-2.0.6), and the parameters are: minimum contig length = 200, min_kmer_cov = 2, and min_glue = 3 [23]. All contigs were generated by merging the sequences with a certain overlap length. The paired-end reads were then mapped back to the contigs, and the distance between the two end reads was revealed. Subsequently, the contigs were connected by the Trinity assembler to obtain the sequences that could no longer be extended on either end. These sequences were referred to as unigenes. The resulting unigenes were then subjected to sequence splicing and redundancy elimination by using the TGICL software system (Linux x86) with the parameters of repeats tringency = 0.95, min_match = 35, and min_score = 35 [24] in order to yield non-redundant unigenes with the maximum length. As a metric for assembly quality, the distribution of contigs and length of unigenes were calculated. After gene family clustering, all unigenes assigned into 2 categories: (i) cluster (unigenes with 70% similarity to each other) and (ii) singleton. Finally, the sequence directions of the unigenes were evaluated.

### 2.4. Functional Annotation

To characterize their putative functions, all unigenes were first aligned with the *NCBI* non-redundant (NR) protein database, *Swiss-Prot Protein* database, *Cluster of Orthologous Groups of Proteins* (COG) database, and *Kyoto Encyclopedia of Genes and Genomes* (KEGG) pathway enrichment database by using BLASTx with a threshold E-value of 1.0 × 10^−5^. The unigenes were then aligned with *the non-redundant nucleotide* (NT) database in GenBank by BLASTn with an E-value of 1.0 × 10^−5^. According to a *gene ontology* (GO) functional classification, the unigenes were annotated using Blast2GO against the NCBI-NR database with a threshold E-value of 1.0 × 10^−5^ [25]. To assess the distribution of gene functions in *P. chinensis* at the macro level, GO functional classification (molecular function, cellular component, and biological process) was conducted for all unigenes using the WEGO software program [26]. KEGG, an important public pathway-related database [27], was employed to unravel the complex functions of the unigenes associated with flower development in *P. chinensis*. To examine the difference in gene expression between male and female buds, the transcription levels of unigenes were quantified by aligning the RNA-seq reads from each library to the assembly. *p*-value < 0.01, FDR ≤ 0.001, and log2 (fold change) ≥ 1 or ≤−1 were used as thresholds to determine the significant differences between two samples.

### 2.5. Simple Sequence Repeat Discovery and Primer Design

SSR motifs were screened using a microsatellite program MISA) [28]. The SSR loci with at least 12 repeats for mononucleotide repeats, 6 for dinucleotide repeats, 5 for trinucleotide and quadnucleotide repeats, and 4 for pentanucleotide and hexanucleotide repeats were selected for further analysis. Primer3 (http://bioinfo.ut.ee/primer3 10 May 2022) [29] was used to design the PCR primers based on the following criteria: (i) a primer length of 18–28, optimal = 23 bases; (ii) PCR product size of 80–300 bp, optimal = 80–160 bp; (iii) GC content of 40–70%, optimal = 50%; and (iv) primer annealing temperature of 55–65 °C with ≤2 °C difference between forward and reverse primers, optimal = 60 °C.

### 2.6. DNA Extraction and EST-SSR Marker Evaluation

Leaf samples of 100 female and 102 male *P. chinensis* trees were collected from Anyang, Henan Province, China. After drying, genomic DNA was isolated from each leaf sample using the cetyltrimethylammonium bromide (CTAB) extraction method [30]. The quality and quantity of DNA samples were evaluated using the NanoDrop 2000 spectrophotometer. To construct two DNA pools using bulked segregant analysis (*BSA*), 20 DNA samples in each group of females and males were equally mixed. Then, the concentration of each DNA sample was diluted to 50 ng/μL, and all samples were kept at −20 °C until further analysis. Polymerase chain reaction (PCR) and gel electrophoresis were carried out to evaluate the amplification of 151 SSR primer pairs. PCR assay was initiated with 10 μL of reaction mixture containing 0.1 μL of Taq polymerase (5 U/μL), 1.0 μL of 10× PCR buffer with MgCl_2_ (25 mM), 0.8 μL of dNTPs (2.5 mmol/L), 0.2 μL of each primer (10 mmol/L), 2.0 μL of template DNA (50 ng/mL), and 5.9 μL of sterile distilled water. PCR conditions were set as follows: an initial denaturation of 94 °C for 3 min, followed by 35 cycles of 94 °C for 30 s, 55–65 °C for 45 s, and 72 °C for 1 min and a final extension of 72 °C for 5 min. Equivalent aliquots (6 μL) of the PCR products were electrophoresed on a 6% polyacrylamide gel. After electrophoresis at 2000 V for 1–1.5 h, the PCR bands were visualized by silver nitrate staining.

## 3. Results

### 3.1. Illumina Paired-End Sequencing and De Novo Assembly of P. chinensis Transcriptome

In total, 54,895,796 and 55,210,442 sequence reads were generated from the female and male buds, respectively. Of these, 50,925,088 and 51,470,578 sequences were of high quality after filtering (Table 1). Considering that there is no reference genome available for *P. chinensis*, the high-quality sequences obtained from the two cDNA libraries were integrated into a reference transcriptome by de novo assembly using the Trinity short reads assembler [23].

**Figure 2:**
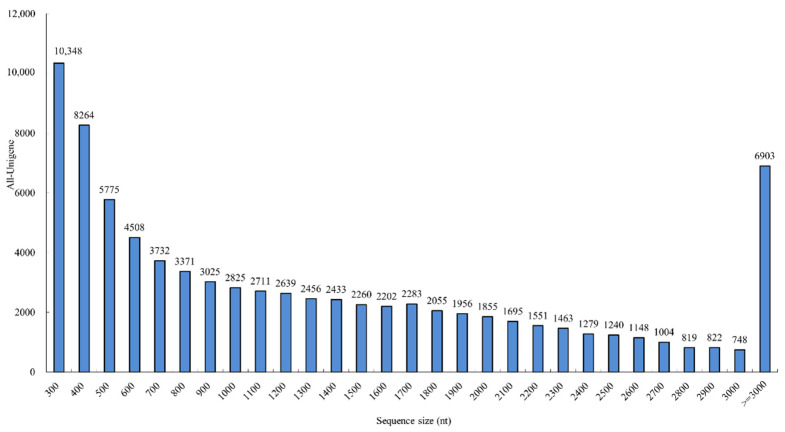
Sequence length distribution of the unigenes in *P. chinensis*.

De novo assembly yielded 83,370 unigenes with a mean length of 1325 bp, N50 value of 2027 bp, and total length of over 11.05 Gb. There were 24,387 unigenes (29.25%) ranging from 201 to 500 bp in length, 17,461 unigenes (20.94%) ranging from 501 to 1000 bp, 12,499 unigenes (14.99%) ranging from 1001 to 1500 bp, 10,351 unigenes (12.42%) ranging from 1501 to 2000 bp, and 18,672 unigenes (22.40%) with >2000 bp long (Table 2 and Figure 2). These findings indicated that the assembled sequences were qualified for subsequent analyses.

### 3.2. Sequence Annotation of P. chinensis Transcriptome

The assembled sequences were annotated with the above-mentioned databases (E-value < 10^−5^), including *COG*, *GO*, *KEGG*, *NR*, *NT*, and *Swiss-Prot*. As shown in Table 3, 64,539 (77.48%) of the 83,370 assembled sequences were annotated, including 59,316 (71.15%) in *NT*, 58,543 (70.22%) in *NR*, 47,049 (56.43%) in *COG*, 40,643 (48.75%) in *GO*, 38,879 (46.63%) in *Swiss-Prot*, and 36,136 (43.34%) in *KEGG*. The 1279 unigenes with no alignment hit in *Swiss-Prot* or *NR* database were further analyzed by ESTS can version 3.0.2 [31]. In total, 57,502 homologous unigenes were identified from both *Swiss-Prot* and *NR* databases or ESTS can program.

**Figure 3:**
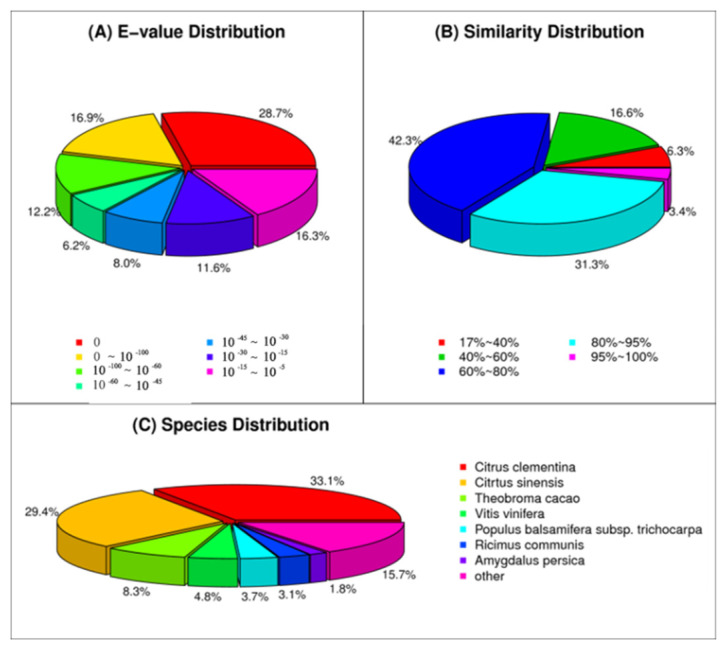
Characterization of the homologous sequences of *P. chinensis* unigenes blasted against the non-redundant database. (**A**) Frequency distribution of the unigene sequences according to their E values (cut-off value = 1.0 × 10^−5^). (**B**) Percentage of the top matched unigene sequences in *P. chinensis*. (**C**) Species distribution of the matched homologous sequences with an E-value of 1.0 × 10^−5^.

As shown in Figure 3A, the majority of the unigene sequences (72.17%) exhibited high homology (E-value < 10^30^) with the publicly available plant sequences, 64.12% had extremely high homology (E-value < 10^−45^), and 27.82% had moderately high homology (E-value = 10^−30^–10^−5^). The analysis of similarity distribution revealed that 3716 (6.35%), 9737 (16.63%), 24,753 (42.28%), 18,341 (31.33%), and 1996 (3.41%) sequences were 17–40, 41–60, 61–80, 81–95, and 95–100% similar, respectively (Figure 3B). For species distribution, 33.15%, 29.45%, and 8.30% of the unigene sequences were matched to those of *Citrus clementina*, *Citrtus sinensis*, and *Theobroma cacao*, respectively. The proportion of unigenes matched with *Vitis vinifera*, *Populus balsamifera*, *Ricimus communis* and *Amygdalus persica* was lower than 5%, while 15.72% of the distinct sequences were similar to those of “other” species (Figure 3C).

**Figure 4:**
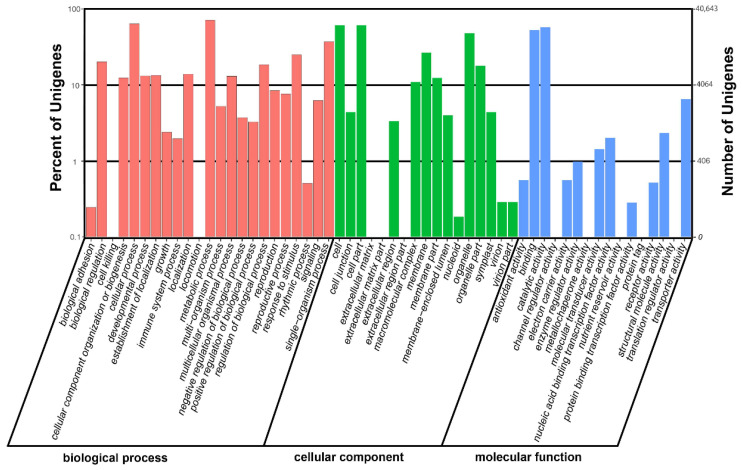
Gene ontology annotations of the assembled unigenes.

The results of *NR* annotation demonstrated that 40,643 unigenes were assigned to at least one *GO* term. The sequences enriched in “molecular function”, “cellular component”, and “biological process” clusters were grouped into 46 functional groups (Figure 4). The most dominant groups of the three major clusters were “cellular processes” and “metabolic processes”, “cell” and “cell part”, and “binding” and “catalytic activity”, respectively (Figure 4).

**Figure 5:**
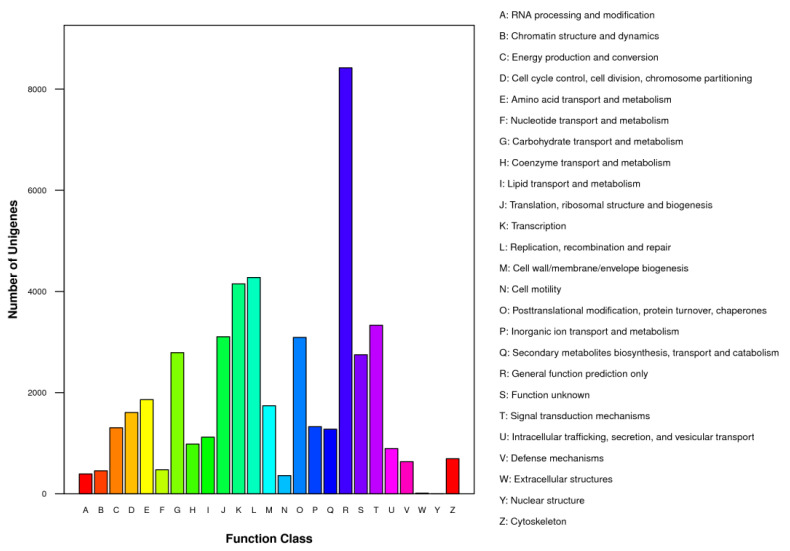
Results for the clusters of orthologous groups classification.

Furthermore, all unigenes were searched against the *COG* database for functional classification. In total, 47,049 of the 58,543 unigenes were assigned to 25 *COG* categories, including biochemistry metabolism, cellular structure, molecular processing, and signal transduction (Figure 5). Of all clusters, “general function prediction only” (8420, 17.90%) was the most dominant, followed by “replication, recombination, and repair” (4276, 9.09%), and “transcription” (4149, 8.82%). However, only 13 and 2 unigenes were classified under “extracellular structure” and “nuclear structure”, respectively (Figure 5). 

### 3.3. KEGG Pathway Assignment of Unigenes

Overall, 36,136 assembled sequences were assigned to 128 KEGG pathways, ranging from 3 to 8208 for each pathway. Table 4 shows the top 20 pathways with the highest sequence numbers. The most abundant genes were assigned to “metabolic pathways” (8208, 22.71%), followed by “biosynthesis of secondary metabolites” (4045, 11.19%), “plant–pathogen interaction” (2516, 6.96%), and “plant hormone signal transduction” (1729, 4.78%). More importantly, some unigenes were also enriched in KEGG pathways related to metabolism, including “metabolic pathways” and “biosynthesis of secondary metabolites”. All these pathways can play a key role in metabolic regulation.

### 3.4. Molecular Characterization of SSR Motifs

A total of 21,662 mononucleotide, dinucleotide, trinucleotide, tetranucleotide, pentanucleotide, and hexanucleotide SSR motifs (12, 6, 5, 5, 4, and 4 repeat numbers, respectively) were detected, and the mononucleotide motif was the most dominant type of repeats (52.59%) in *P. chinensis*, followed by dinucleotide (22.29%), trinucleotide (20.15%), hexanucleotide (2.28%), pentanucleotide (1.51%), and tetranucleotide motifs (1.18%). As shown in Table 5 and (Supplementary Table S1), the most abundant repeats were AG/CT (13.97%), followed by AAC/GTT (6.75%), AT/TA (6.10%), AAT/TAA (2.90%), and ACC/GGT (2.61%). The distribution of SSR motifs in *P. chinensis* was calculated as one SSR per 5.10 kb.

### 3.5. Identification of Sex-Linked SSR Markers

**Figure 6:**
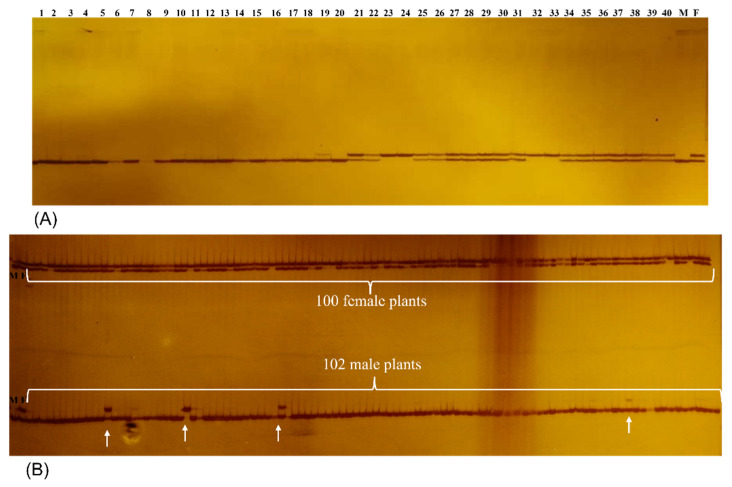
Electrophoresis-based visualization of PCR bands amplified with the EST-SSR primer pair PcSSR51. Lanes F and M denote female bulk and male bulk, respectively. (**A**) Lanes 1–20 and 21–40 represent the PCR products of male and female *P. chinensis*, respectively. (**B**) Lanes with white braces 1–102 indicate the PCR products of male *P. chinensis*; lanes with white braces 1–100 represent the PCR products of female *P. chinensis*; and white arrowheads denote the female-specific EST-SSR marker amplified with PcSSR55 primer pair.

Among the 983 primer pairs, 151 were randomly selected to screen the efficiency of these primers and to identify sex-linked EST-SSR markers in *P. chinensis* (Supplementary Table S2). Of the selected primers, 138 (91.39%) pairs showed clear amplifications in *P. chinensis*, whereas the remaining 13 failed to amplify. In the genotyping assay combined with BSA, all the 151 primer pairs were employed to detect putative EST-SSR markers in two DNA bulks of female and male *P. chinensis*. Notably, 25 primer pairs showed distinct polymorphisms between the two DNA bulks. To evaluate whether these polymorphisms can be used for sex identification, 20 DNA samples in each group of females and males were detected separately. The results showed that only PcSSR55 produced a female-specific marker that was not detectable in all male DNA samples (Figure 6A). To further verify the female-specific marker, 100 female and 102 male DNA samples were amplified with PcSSR55 primer pair, and the results showed that all the female DNA samples exhibited the specific marker band, and only four male samples showed amplification of the specific band (Figure 6B). Altogether, these findings indicate that PcSSR55 is effective for the sex determination of *P. chinensis*.

## 4. Discussion

### 4.1. Characterization of P. chinensisTranscriptome

Next-generation sequencing technology presents opportunities for plant genome analysis and offers a fast, cost-effective way to characterize the whole transcriptomes of various organisms [32]. This technique has been applied to sequence an array of non-model plants, including strawberry, pistachio, grasspea, and others [33,34,35,36]. In this work, the pooled RNA samples from female and male *P. chinensis* were analyzed by the Illumina RNA-seq platform, and de novo assembly of their transcriptomes was conducted due to the scarcity of reference sequences in the publicly available databases. The quality of a de novo assembly can be assessed by the mean length and N50 value of the contigs. As presented in Table 2, the mean length and N50 value of the unigenes were 1.3 and 2.0 kb, respectively, which were comparatively better than other transcriptomic studies [37,38,39] and other *Pistacia* transcriptomes published in the literature [40,41]. Although the higher N50 value and greater average length can indicate an accurate and effective assembly [37,42], previous research has suggested that both measures are primitive and often misleading [43]. Thus, it is generally believed that N50 can be applied to measure the continuity of the unigenes but not their applicability [44].

Of the 83,370 high-quality unigenes of *P. chinensis* transcriptome, 64,539 unigenes (77.48%) were successfully annotated to the six databases (i.e., *COG*, *GO*, *KEGG*, *NR*, *NT*, and *Swiss-Prot*), and only 22.52% unigenes did not significantly match to any of those six datasets, which might be attributed to their short full-length transcripts or the high threshold of E-value [45]. Moreover, it is speculated that these unmatched unigenes have no similar annotations in the six databases and may represent the species-specific genes without prior characterization. From the BLASTX search against the *NR* database, 84.28% of the identified unigenes of *P. chinensis* displayed high homology with those of *C. clementina*, *C. sinensis*, *T. cacao*, *V. vinifera*, *P. balsamifera* subsp., *trichocarpa*, *R.* *communis*, and *A. persica* (Figure 3). Such similarity might be due to the lack of whole-genome-sequencing data in the publicly available databases for the related *Pistacia* species. Through the use of COG and GO databases, the unigenes were categorized into 25 sub-terms and 46 sub-categories (Figure 4 and Figure 5), demonstrating that the identified unigenes possess a wide range of important functions in *P. chinensis* [46,47]. Next, a total of 36,136 unigenes were annotated and mapped to 128 KEGG pathways (Table 3), which help us to reveal the metabolic pathways and gene interaction. In addition, 1729 unigenes involved in “plant hormone signal transduction” were identified, and these findings may help us to discover potential candidate genes related to the sex differentiation of *P. chinensis* in the future. In short, de novo RNA-seq based transcriptome analysis of *P. chinensis* can facilitate future studies on the physiological, biochemical, and molecular aspects of other *Pistacia* species.

### 4.2. Abundance and Distribution of SSR Motifs

Transcriptome sequencing has been commonly used to screen SSRs in various angiosperm species, including *Fragaria* × *Potentilla* (red-flowering strawberry), *Amentotaxus argotaenia*, *Curcuma alismatifolia*, *Vigna angularis*, *P. vera,* and *P. chinensis* [22,33,37,38,39,40,41]. In this study, out of 83,370 unigenes, 17,028 unigenes consisted of SSR motifs, accounting for 20.42% of total sequences, with a SSR distribution density of 1 per 5.1 kb (Table 4 and Table 5). These values were relatively comparable to those of *Arachi shypogaea* (17.7%, 3.3 kb) [48], *Cyamopsis tetragonoloba* (13.3%, 9.8 kb) [49], *C. alismatifolia* (12.57%, 6.6 kb) [38], *Fragaria* × *Potentilla* (red-flowered strawberry) (10.24%, 10.7 kb) [33], *Torreya grandis* (2.7%, 25.9 kb) [50], and *Zingiber officinale* (2.7%, 25.2 kb) [51]. Such divergence in the abundance and distribution of SSR motifs among various plant species could be partially explained by the variations between species, SSR search criteria, size of the datasets, sequence redundancy, and types of data mining tools [31,36,52]. The nucleotide characteristics of SSRs may be varied in different plants, and trinucleotides showed the highest repetition rate in *Amorphophallus konjac* and *A. bulbifer* [53], *C. alismatifolia* [38], *Actinidia chinensis* [54], *Z. officinale* [51], and *P. chinensis* [22]. In contrast, our results demonstrated that dinucleotide SSR was the most abundant type, which were consistent with those of previous studies conducted on *V. angularis* [37], *Idesia polycarpa* [36], red-flowering strawberry [33], and *P. vera* [41]. Additionally, the most common dinucleotide repeat in *P. chinensis* was AG/CT motif (13.97%), similar to that reported in *P. vera* [41], *C. alismatifolia* [37], *Brassica napus* [30], *V. angularis* [37], and *I. polycarpa* [36], followed by AT/TA (6.09%) and AC/GT (2.21%). Furthermore, GC/CG repeat was rarely found in eukaryotic genomes, and this might be the case for *P. chinensis* [23]. The most frequent trinucleotide repeat motifs observed in *P. chinensis* were in the following order: AAG/CTT (6.75%), AAT/ATT (2.90%), and ACC/GGT (2.61%). Similar findings were also reported in *V. angularis* [34], *B. napus* [27], *I. polycarpa* [36], and *A. argotaenia* [39]. Taken together, these data indicate that the trinucleotide motif AAG/CTT is common in *P. chinensis* [22]. Besides, we also noticed that AT-rich trinucleotide motifs (AAG/CTT, AAT/ATT, AAC/GTT, ACT/AGT, and ATC/ATG > 71%) were more abundant than GC-rich trinucleotide (ACC/GGT, AGG/CCT, AGC/GCT, ACG/CGT, and CCG/CGG, <29%). These data suggest that the SSR motif of *P. chinensis* is more toward AT-rich [22].

### 4.3. Validation and Polymorphism of EST-SSRs for Gender Identification of P. chinensis

Among the agriculturally important crops, such as pistachio, papaya, kiwifruit, and date palm, female trees are responsible for the production of commercial crop [55]. Thus, it is crucial to identify the gender of these plants [41]. For the development of EST-SSR markers, transcriptomic data mining can provide greater efficiency and flexibility in biomarker selection. SSR marker detection possesses several distinct advantages over other techniques, including low cost, rapidity, and being commonly available and applicable in various plants such as *Myrica rubra* [56], *Phoenix dactylifera* [57], *A. chinensis* [54], and *Tapiscia sinensis* [58]. In this study, 151 EST-SSR markers were developed and validated, which could provide necessary information for the determination of sex-linked markers in *P. chinensis*. Among these markers, one SSR marker was able to distinguish between female and male *P.* *chinensis*, and the percentage of accurate identification of sex was more than 98%. However, only 2% of the individual gender could not be identified. The reason may be that there is a certain physical distance on the genome between the marker and the gene controlling female traits, so the marker is only closely linked to the female and cannot be co-separated with the female. Previous studies also found that it was not easy to develop sex-linked markers in *P. vera* with 100% accuracy [5,6,7]. Until the male and female progenies belonging to the Siirt × Bağyolu F_1_ population were sequenced, eight loci from seven RAD reads were successfully able to distinguish the sexes in *P. vera* [8]. Furthermore, seven novel sex-linked SNP markers were identified and mapped to the center of the chromosome and were therefore considered potential sex-linked markers for MAS in *P. vera* [9].

## 5. Conclusions

From the transcriptomic analysis of female and male *P. chinensis*, 83,370 unigenes were de novo assembled, and 77.48% of them were annotated and mapped to the publicly available databases. In addition, 21,662 SSR motifs were characterized, and one SSR marker was specific to female *P. chinensis*. Altogether, our findings provide useful insights into the genetic mechanism underlying sex differences in *P. chinensis*, which can be used as the basis for further research on the functional genomics and reproductive biology of this plant. Furthermore, the established EST-SSR markers may serve as an important molecular tool for the conservation and MAS of *P. chinensis*.

## Figures and Tables

**Table 1:** Raw data output of Illumina RNA sequencing.

Item	Total Raw Reads	Total Clean Reads	Total Clean Nucleotides (nt)	Q20	Q30	GC Content
PC	54,895,796	50,925,088	7638,763,200	98.29%	96.56%	43.52%
PX	55,210,442	51,470,578	7720,586,700	98.25%	96.43%	43.43%

Note: PC mean female bulk and PX mean male bulk. Q20 and Q30 percentages are the proportion of nucleotides with quality value larger than 20 and 30, respectively; GC percentage is the proportion of guanine and cytosine nucleotides among total nucleotides.

**Table 2:** Quality of the assembled RNA sequencing data.

Item	Total Number (nt)	Total Length (nt)	Mean Length (nt)	N50	Distinct Clusters	Distinct Singletons
PC_Contigs	89,442	93,938,149	1050	1739		
PX_Contigs	89,724	96,205,186	1072	1768		
PC_Unigenes	65,520	80,247,826	1225	1903	29,689	35,831
PX_Unigenes	65,752	82,513,165	1255	1933	30,903	34,849
All_Unigenes	83,370	110,503,948	1325	2027	42,960	40,410

**Table 3:** Summary of the annotated and assembled sequences of *P. chinensis*.

Sequence File	*NR*	*NT*	*Swiss-Prot*	*KEGG*	*COG*	*GO*	All Annotated Unigenes	All Assembled Unigenes
Number of Unigenes (singleton, cluster, unigene)	58,543 (20,307, 38,236)	59,316 (21,071, 38,245)	38,879 (13,566, 25,313)	36,136 (12,125, 24,011)	47,049 (13,999, 33,050)	40,643 (12,965, 27,678)	64,539 (24,634, 39,905)	83,370 (40,410, 42,960)
Annotated/All-Unigene (%)	70.22	71.15	46.63	43.34	56.43	48.75	77.48	

**Table 4:** Top 20 pathways with highest sequence numbers.

Rank	Pathway	Genes within the Coverage of Pathway Annotations (*n* = 36,136)	Pathway ID
1	Metabolic pathways	8208 (22.71%)	ko01100
2	Biosynthesis of secondary metabolites	4045 (11.19%)	ko01110
3	Plant-pathogen interaction	2516 (6.96%)	ko04626
4	Plant hormone signal transduction	1729 (4.78%)	ko04075
5	Spliceosome	1336 (3.7%)	ko03040
6	RNA transport	1203 (3.33%)	ko03013
7	Pyrimidine metabolism	1096 (3.03%)	ko00240
8	Purine metabolism	1086 (3.01%)	ko00230
9	Ribosome	981 (2.71%)	ko03010
10	Protein processing in endoplasmic reticulum	962 (2.66%)	ko04141
11	Endocytosis	811 (2.24%)	ko04144
12	Starch and sucrose metabolism	757 (2.09%)	ko00500
13	Ubiquitin mediated proteolysis	746 (2.06%)	ko04120
14	RNA polymerase	719 (1.99%)	ko03020
15	Ribosome biogenesis in eukaryotes	684 (1.89%)	ko03008
16	Glycerophospholipid metabolism	677 (1.87%)	ko00564
17	RNA degradation	648 (1.79%)	ko03018
18	mRNA surveillance pathway	600 (1.66%)	ko03015
19	Phenylpropanoid biosynthesis	574 (1.59%)	ko00940
20	Glycolysis/Gluconeogenesis	530 (1.47%)	ko00010

**Table 5:** Sequence searching for the SSR markers of *P. chinensis*.

Searching Item	Numbers
Total number of examined sequences	83,370
Total size of examined sequences (bp)	110,503,948
Total number of identified SSR markers	21,662
Number of SSR-containing sequences	17,028
Number of sequences with >1 SSR	3545
Number of SSR markers found in compound formation	1376

## Data Availability

All Illumina clean data generated for this study were deposited in the CNGB Sequence Archive (CNSA: https://db.cngb.org/cnsa/, 10 May 2022) of CNGBdb with accession number CNP0001207(2020-07-30), while the assembled transcriptomes have been deposited in CNGB (CNP0001474)(2020-12-11).

## Supplementary Materials

TThe following supporting information can be downloaded at: https://www.mdpi.com/article/10.3390/genes13061024/s1, Table S1: Primers sequences for the 151 EST-SSR markers of *P. chinensis*; Table S2 Primers sequences of 151 EST-SSR markers for *P. chinensis*.

## References

[1] Karmakar A., Karmakar S., Mukherjee S. (2010). Properties of various plants and animals feed stocks for biodiesel production. Bioresour. Technol..

[2] Kafkas S., Özkan H., Ak B.E., Acar I., Atli H.S., Koyuncu S. (2006). Detecting DNA polymorphism and genetic diversity in a wide pistachio germplasm: Comparison of AFLP, ISSR and RAPD markers. J. Am. Soc. Hortic. Sci..

[3] Qin S.J., Sun Y.Z., Meng X.C., Zhang S.X. (2010). Production and analysis of biodiesel from non-edible seed oil of *Pistacia chinensis*. Energy Explor. Exploit..

[4] Wang L.B., Yu H.Y., He X.H. (2012). Assessment on fuel properties of four woody biodiesel plants species in China. Sci. Silvae Sin..

[5] Hormaza J.I., Dollo L., Polito V.S. (1994). Identification of a RAPD marker linked to sex determination in *Pistacia vera* using bulked segregant analysis. Theor. Appl. Genet..

[6] Yakubov B., Barazani O., Golan-Goldhirsh A. (2005). Combination of SCAR primers and touchdown-PCR for sex identification in *Pistacia vera* L. Sci. Hortic..

[7] Esfandiyari B., Davarynejad G.H., Shahriari F., Kiani M., Mathe A. (2012). Data to the sex determination in *Pistacia* species using molecular markers. Euphytica.

[8] Kafkas S., Khodaeiaminjan M., Güney M., Kafkas E. (2015). Identification of sex-linked SNP markers using RAD sequencing suggests ZW/ZZ sex determination in *Pistacia vera* L. BMC Genom..

[9] Khodaeiaminjan M., Kafkas E., Güney M., Kafkas S. (2017). Development and linkage mapping of novel sex-linked markers for marker-assisted cultivar breeding in pistachio (*Pistacia vera* L.). Mol. Breed..

[10] Sun Q., Yang X., Li R. (2014). SCAR marker for sex identification of *Pistacia chinensis* Bunge (Anacardiaceae). Genet. Mol. Res..

[11] Feng C., Chen M., Xu C.J., Bai L., Yin X.R., Li X., Allan A.C., Ferguson I.B., Chen K.S. (2012). Transcriptomic analysis of Chinese bayberry (*Myrica rubra*) fruit development and ripening using RNA-Seq. BMC Genom..

[12] Fu B.D., He S.P. (2012). Transcriptome analysis of silver carp (*Hypophthalmichthys molitrix*) by paired-end RNA sequencing. DNA Res..

[13] Brautigam A., Mullick T., Schliesky S., Weber A. (2011). Critical assessment of assembly strategies for non-model species mRNA-Seq data and application of next-generation sequencing to the comparison of C (3) and C (4) species. J. Exp. Bot..

[14] Hahn D.A., Ragland G.J., Shoemaker D.D., Denlinger D.L. (2009). Gene discovery using massively parallel pyrosequencing to develop ESTs for the flesh fly *Sarcophaga*
*crassipalpis*. BMC Genom..

[15] Xiang L.X., He D., Dong W.R., Zhang Y.W., Shao J.Z. (2010). Deep sequencing-based transcriptome profiling analysis of bacteria-challenged *Lateolabrax*
*japonicus* reveals insight into the immune-relevant genes in marine fish. BMC Genom..

[16] Sierocka I., Alaba S., Jarmolowski A., Karlowski W.M., Szweykowska-Kulinska Z. (2020). The identification of differentially expressed genes in male and female gametophytes of simple thalloid liverwort *Pellia endiviifolia* sp. B using an RNA-seq approach. Planta.

[17] Ramos M.J.N., Coito J., Fino J., Cunha J., Silva H., de Almeida P.G., Costa M.M.R., Amancio S., Paulo O.S., Rocheta M. (2017). Deep analysis of wild Vitis flower transcriptome reveals unexplored genome regions associated with sex specification. Plant Mol. Biol..

[18] Prentout D., Razumova O., Rhone B., Badouin H., Henri H., Feng C., Kafer J., Karlov G., Marais G.A.B. (2020). An efficient RNA-seq-based segregation analysis identifies the sex chromosomes of *Cannabis sativa*. Genome Res..

[19] Gao P., Sheng Y.Y., Luan F.S., Ma H.Y., Liu S. (2015). RNA-Seq transcriptome profiling reveals differentially expressed genes involved in sex expression in melon. Crop Sci..

[20] Xin G.L., Liu J.Q., Liu J., Ren X.L., Du X.M., Liu W.Z. (2019). Anatomy and RNA-Seq reveal important gene pathways regulating sex differentiation in a functionally Androdioecious tree, *Tapiscia sinensis*. BMC Plant Biol..

[21] Powell W., Morgante M., Andre C., Hanafey M., Vogel J., Tingey S., Rafalski A. (1996). The comparison of RFLP, RAPD, AFLP and SSR (microsatellite) markers for germplasm analysis. Mol. Breed..

[22] Choi K.Y., Park D.H., Seong E.S., Sang W.L., Na J.K. (2019). Transcriptome analysis of a medicinal plant, *Pistacia chinensis*. J. Plant Biotechnol..

[23] Grabherr M.G., Haas B.J., Yassour M., Levin J.Z., Thompson D.A., Amit I., Adiconis X., Fan L., Raychowdhury R., Zeng Q. (2011). Full-length transcriptome assembly from RNA-Seq data without a reference genome. Nat. Biotechnol..

[24] Pertea G., Huang X.Q., Liang F., Antonescu V., Sultana R., Karamycheva S., Lee Y., White J., Cheung F., Parvizi B. (2003). TIGR Gene Indices clustering tools (TGICL): A software system for fast clustering of large EST datasets. Bioinformatics.

[25] Conesa A., Götz S., García-Gómez J.M., Terol J., Talón M., Robles M. (2005). Blast2GO: A universal tool for annotation, visualization and analysis in functional genomics research. Bioinformatics.

[26] Ye J., Fang L., Zheng H.K., Zhang Y., Chen J., Zhang Z.J., Wang J., Li S.T., Li R.Q., Bolund L. (2006). WEGO: A web tool for plotting GO annotations. Nucleic Acids Res..

[27] Kanehisa M., Araki M., Goto S., Hattori M., Hirakawa M., Itoh M., Itoh M., Katayama T., Kawashima S., Okuda S. (2008). KEGG for linking genomes to life and the environment. Nucleic Acids Res..

[28] Thiel T., Michalek W., Varshney R.K., Graner A. (2003). Exploiting EST databases for the development and characterization of gene-derived SSR-markers in barley (*Hordeum vulgare* L.). Theor. Appl. Genet..

[29] Koressaar T., Lepamets M., Kaplinski L., Raime K., Andreson R., Remm M. (2018). Primer3_masker: Integrating masking of template sequence with primer design software. Bioinformatics.

[30] Cheng X.M., Xu J.S., Xia S., Gu J.X., Yang Y., Fu J., Qian X.J., Zhang S.C., Wu J.S., Liu K.D. (2009). Development and genetic mapping of microsatellite markers from genome survey sequences in *Brassica napus*. Theor. Appl. Genet..

[31] Iseli C., Jongeneel C.V., Bucher P. ESTScan: A program for detecting, evaluating, and reconstructing potential coding regions in EST sequences. Proceedings of the Seventh International Conference on Intelligent Systems for Molecular Biology.

[32] Wei Z., Sun Z., Cui B., Zhang Q., Xiong M., Wang X., Zhou D. (2016). Transcriptome analysis of colored calla lily (*Zantedeschia rehmannii* Engl.) by Illumina sequencing: De novo assembly, annotation and EST-SSR marker development. PeerJ.

[33] Ding Y., Xue L., Guo R.X., Luo G.J., Song Y.T., Lei J.J. (2019). De Novo assembled transcriptome analysis and identification of genic SSR markers in red-flowered strawberry. Biochem. Genet..

[34] Zeng L., Tu X.L., Dai H., Han F.M., Lu B.S., Wang M.S., Nanaei H.A., Tajabadipour A., Mansouri M., Li X.L. (2019). Whole genomes and transcriptomes reveal adaptation and domestication of pistachio. Genome Biol..

[35] Hao X.P., Yang T., Liu R., Hu J.G., Yao Y., Burlyaeva M., Wang Y., Ren G.X., Zhang H.Y., Wang D. (2017). An RNA sequencing transcriptome analysis of Grasspea *(Lathyrus*
*sativus* L.) and development of SSR and KASP markers. Front. Plant Sci..

[36] Mei L., Dong N., Li F., Li N., Yao M., Chen F., Tang L. (2017). Transcriptome analysis of female and male flower buds of *Idesia*
*polycarpa* Maxim. var. vestita Diels. Electron. J. Biotechnol..

[37] Chen H.L., Wang L.L., Wang S.H., Somta P., Cheng X.Z. (2015). Development and Validation of EST-SSR Markers from the Transcriptome of Adzuki Bean (*Vigna angularis*). PLoS ONE.

[38] Taheri S., Abdullah T.L., Rafii M.Y., Harikrishna J.A., Werbrouck S.P.O., Teo C.H., Sahebi M., Azizi P. (2019). De novo assembly of transcriptomes, mining, and development of novel EST-SSR markers in *Curcuma alismatifolia* (Zingiberaceae family) through Illumina sequencing. Sci. Rep..

[39] Ruan X., Wang Z., Wang T., Su Y. (2019). Characterization and application of EST-SSR markers developed from the transcriptome of *Amentotaxus argotaenia* (Taxaceae), a relict vulnerable conifer. Front. Genet..

[40] Dong S.B., Liu Y.L., Xiong B., Jiang X.N., Zhang Z.X. (2016). Transcriptomic analysis of a potential bioenergy tree, *Pistacia chinensis* Bunge, and identification of candidate genes involved in the biosynthesis of oil. Bioenergy Res..

[41] Moazzzam Jazi M., Mahdi Seyedi S., Ebrahimie E., Ebrahimi M., De Moro G., Botanga C. (2017). A genome-wide transcriptome map of pistachio (*Pistacia vera* L.) provides novel insights into salinity-related genes and marker discovery. BMC Genom..

[42] Li X., Li M., Hou L., Zhang Z.Y., Li Y.Y. (2018). De novo transcriptome assembly and population genetic analyses for an endangered chinese endemic *Acer miaotaiense* (Aceraceae). Genes.

[43] Li B., Fillmore N., Bai Y.S., Collins M., Thomson J.A., Stewart R., Dewey C.N. (2014). Evaluation of de novo transcriptome assemblies from RNA-Seq data. Genome Biol..

[44] Salzberg S.L., Phillippy A.M., Zimin A., Puiu D., Magoc T., Koren S., Treangen T.J., Schatz M.C., Delcher A.L., Roberts M. (2012). GAGE: A critical evaluation of genome assemblies and assembly algorithms. Genome Res..

[45] Parchman T.L., Geist K.S., Grahnen J.A., Benkman C.W., Buerkle C.A. (2010). Transcriptome sequencing in an ecologically important tree species: Assembly, annotation, and marker discovery. BMC Genom..

[46] Zhou T., Li Z.H., Bai G.Q., Feng L., Chen C., Wei Y., Chang Y.X., Zhao G.F. (2016). Transcriptome sequencing and development of genic SSR markers of an endangered Chinese endemic genus *Dipteronia oliver* (Aceraceae). Molecules.

[47] Yang Y.X., Chen X.X., Xu B., Li Y.X., Ma Y.H., Wang G.D. (2015). Phenotype and transcriptome analysis reveals chloroplast development and pigment biosynthesis together influenced the leaf color formation in mutants of *Anthuriuman draeanum* ‘Sonate’. Front. Plant Sci..

[48] Wang H.M., Lei Y., Yan L.Y., Wan L.Y., Cai Y., Yang Z.F., Lv J.W., Zhang X.J., Xu C.W., Liao B.H. (2018). Development and validation of simple sequence repeat markers from *Arachi shypogaea* transcript sequences. Crop. J..

[49] Al-Qurainy F., Alshameri A., Gaafar A.R., Khan S., Nadeem M., Alameri A.A., Tarroum M., Ashraf M., Kurabayashi A. (2019). Comprehensive stress-based De Novo transcriptome assembly and annotation of guar (*Cyamopsis tetragonoloba* (L.) Taub.): An important industrial and forage crop. Int. J. Genom..

[50] Zeng J., Chen J., Kou Y.X., Wang Y.J. (2018). Application of EST-SSR markers developed from the transcriptome of *Torreya*
*grandis* (Taxaceae), a threatened nut-yielding conifer tree. PeerJ.

[51] Awasthi P., Singh A., Sheikh G., Mahajan V., Gupta A.P., Bedi Y.S., Gandhi S.G. (2017). Mining and characterization of EST-SSR markers for *Zingiber*
*officinale* Roscoe with transferability to other species of Zingiberaceae. Physiol. Mol. Biol. Plants.

[52] Kumpatla S.P., Mukhopadhyay S. (2005). Mining and survey of simple sequence repeats in expressed sequence tags of dicotyledonous species. Genome.

[53] Zheng X.F., Pan C., Diao Y., You Y.N., Yang C.Z., Hu Z.L. (2013). Development of microsatellite markers by transcriptome sequencing in two species of *Amorphophallus* (Araceae). BMC Genom..

[54] Zhang Q., Liu C.Y., Liu Y.F., VanBuren R., Zhong C., Huang H. (2015). High-density interspecific genetic maps of kiwifruit and the identification of sex-specific markers. DNA Res..

[55] Muhammad M., Jaskani M.J., Awan F.S., Ahmad S., Khan I.A. (2016). Development of molecular method for sex identification in date palm (*Phoenix dactylifera* L.) plantlets using novel sex-linked microsatellite markers. 3 Biotech.

[56] Jia H.M., Jiao Y., Wang G.Y., Li Y.H., Jia H.J., Wu H.X., Chai C.Y., Dong X., Guo Y.P., Zhang L.P. (2015). Genetic diversity of male and female Chinese bayberry (*Myrica rubra*) populations and identification of sex-associated markers. BMC Genom..

[57] Cherif E., Zehdi S., Castillo K., Chabrillange N., Abdoulkader S., Pintaud J.C., Santoni S., Salhi-Hannachi A., Glémin S., Aberlenc-Bertossi F. (2013). Male-specific DNA markers provide genetic evidence of an XY chromosome system, a recombination arrest and allow the tracing of paternal lineages in date palm. New Phytol..

[58] Zhou X.J., Wang Y.Y., Xu Y.N., Yan R.S., Zhao P., Liu W.Z. (2015). De novo characterization of flower bud transcriptomes and the development of EST-SSR markers for the endangered tree *Tapiscia sinensis*. Int. J. Mol. Sci..

