# Supplementary material for: Transcriptome Analysis and Identification of a Female-Specific SSR Marker in Pistacia chinensis Based on Illumina Paired-End RNA Sequencing
Source: Genes (Basel). 2022 Jun 7;13(6):1024. doi: 10.3390/genes13061024 (PMC9222763; doi:10.3390/genes13061024)
Supplement: Supplementary file 1 [file genes-13-01024-s001.zip › Table S1.pdf]

Table S1 Size distribution of SSR markers according to the number of repeaters.

Number of repeats	4	5	6	7	8	9	10	11	12	13	14	15	16	17	18	19	≥20	Total
Mono-	0	0	0	0	0	0	0	0	1680	1275	1024	948	838	725	634	520	3749	11393(52.59%)
Di-	0	0	1667	818	684	441	364	286	194	38	53	47	58	44	34	30	70	4829(22.29%)
AC/GT	0	0	168	101	51	36	43	27	22	3	1	3	2	6	1	3	13	480(2.22%)
AG/CT	0	0	977	481	426	277	250	194	135	17	47	32	50	37	26	26	52	3027(13.97%)
AT/TA	0	0	522	236	207	128	71	65	37	18	5	12	6	1	7	1	5	1321(6.09%)
GC/CG	0	0	0	0	0	0	0	0	0	0	0	0	0	0	0	0	0	1
Tri-	0	2429	972	531	273	42	47	25	9	5	10	3	9	4	0	1	4	4364(20.15%)
AAC/GTT	0	248	78	46	16	3	0	0	0	0	0	0	0	0	0	1	0	392
AAG/CTT	0	752	338	174	102	22	33	15	5	4	8	3	4	2	0	0	0	1462(6.75%)
AAT/ATT	0	323	165	89	44	1	3	2	0	0	0	0	0	2	0	0	0	629(2.90%)
ACC/GGT	0	347	110	62	28	2	8	3	4	1	0	0	1	0	0	0	0	566(2.61%)
ACG/CGT	0	48	15	8	0	0	0	0	0	0	0	0	0	0	0	0	0	71
ACT/AGT	0	66	17	10	3	2	0	0	0	0	0	0	0	0	0	0	0	98
AGC/GCT	0	181	46	6	12	2	0	0	0	0	0	0	0	0	0	0	0	247
AGG/CCT	0	135	84	48	22	3	2	2	0	0	0	0	0	0	0	0	0	296
ATC/GAT	0	282	108	85	45	7	1	3	0	0	2	0	4	0	0	0	4	541
CCG/CGG	0	47	11	3	1	0	0	0	0	0	0	0	0	0	0	0	0	62
Tetra-	0	193	55	3	1	1	2	0	0	0	0	0	0	0	0	0	0	255(1.18%)
Petra-	275	37	10	2	3	0	0	0	0	0	0	0	1	0	0	0	0	328(1.51%)
Hexa-	414	28	29	2	5	0	1	2	1	2	0	1	3	0	0	0	5	493(2.28)
Total	689	2687	2733	1356	966	484	414	313	1884	1320	1087	999	909	773	668	551	3828	21662
