# Supplementary material for: Transcriptome Analysis and Identification of a Female-Specific SSR Marker in Pistacia chinensis Based on Illumina Paired-End RNA Sequencing
Source: Genes (Basel). 2022 Jun 7;13(6):1024. doi: 10.3390/genes13061024 (PMC9222763; doi:10.3390/genes13061024)
Supplement: Supplementary file 1 [file genes-13-01024-s001.zip › Table S2.pdf]

Table S2 Primers sequences of of 151 EST-SSR markers for *P. chinensis*

Marker ID	Foward primer	Reverse primer	Repeat motif	Allele size(bp)	Amplified primer
PcSSR1	AGGCTTCAAAATCTTCCAATTTC	GGATAAAATATGTGGGCTTGGTT	AAAAAT(4)	156	Yes
PcSSR2	GAAACACATCACAATTTCTGGGT	TGTTGTAGGATTGCGTTATTGTG	AAAAT(4)	150	Yes
PcSSR3	TCACTGCATTTTCTTTCATTTCA	GAAGAAGATACCCATCAACAAACA	AAAGA(4)	159	Yes
PcSSR4	AATGGAAAGAACAAAAGCAATGA	CCAACATCCTAATCTTCCAGAAA	AAAT(5)	143	Yes
PcSSR5	AAGTTGATGAGCTCACCCATAGA	AAGATATGGAGGTTGTGTCCAGA	AAC(6)	132	Yes
PcSSR6	AAACAAATCATCAAAACACACCC	GGCTATCAAAACTTAGCACATCA	AAG(12)	131	Yes
PcSSR7	CGAAAAGTCGTTTGATCCATAAG	TGTCATCTCCAGATTTTTGCTTC	AAG(6)	139	Yes
PcSSR8	CAGCGGTTGAACTAACAAAACTAA	CCTCTCTTCATACTCTTTCTTCCG	AAG(7)	153	Yes
PcSSR9	AAGTAGGAGGGTCAAGAAAAAGC	TTTAATCCTCCCAAACCTTTCAT	AAGG(6)	131	No
PcSSR10	TTTCAAAGAGGATGAATGGTTTG	TTTGAATCTTGTATGCCTCACAG	AAT(6)	149	Yes
PcSSR11	TTGCTTTAGTTTTCAAGGACCAA	AAACTTACATCAGTCAGCAAGCC	AAT(7)	136	Yes
PcSSR12	AGAGCAATGCGCTCTAATAACAC	GTCGAGATGGGTGATTTCTATTG	AATC(5)	144	Yes
PcSSR13	TCAGTTCATAACATTCTGTCTCTTCTG	CCAAGACTGATAGCAGAATAAGGA	AC(8)	158	Yes
PcSSR14	GATTTGATCTCAGGAATCTCCTCT	AGCAAAGAGGACATGAAAACTGA	ACA(6)	157	Yes
PcSSR15	CTTGGCCTAGACAATGATTCTGA	GTCTCTCTGCCTCCACCTCTAC	ACC(6)	137	Yes
PcSSR16	CACACTCTCCACACTGACAAAAC	GGACCATGTGGTTAGGGATTT	ACC(8)	135	Yes
PcSSR17	CAACCAAAACTCGATCGTTTC	CACCTGAGGCTCACATGTCTT	ACTGCC(4)	141	Yes
PcSSR18	ACACAGACACAAACCCTTGAAAT	ATTAGTGAGGCCAAACAGAGACA	AG(11)	135	Yes
PcSSR19	AGCAAATAAGAGAAACAGGAGCC	TGACTTCTCTCTTTCCCCTAACC	AG(12)	159	Yes
PcSSR20	TCTTCATGTCAATACAAAACGGA	CAGAGGCACCAGAATCTCTTTTA	AG(14)	143	Yes
PcSSR21	TTGGAGATATTGTGAGGTTCTGC	CACTCATTCCAACAACAGAGACA	AG(8)	159	Yes
PcSSR22	GGAGGTAAAGTTGAAGCCAGAAC	TGCTACAAGAACAAGGTCCTCTC	AG(8)	158	Yes
PcSSR23	CATTACGTTCTTGTCCCTGACTC	CATATTAAATCCGTACAACCCCA	AG(8)	153	Yes
PcSSR24	ATGCAGCAACTGGAGATGATAAG	CGTTTCATTGCCTTCAAAACTAA	AG(9)	159	Yes
PcSSR25	ATGCAGATTGTTTGGTGTTTCTT	ATATTATGGAGAAGCAGATGGCA	AG(9)	151	Yes
PcSSR26	GAAGAAGAGGAGGAAGAATTTGG	TACATTCTCACACGGGAACTTTT	AGA(6)	152	Yes
PcSSR27	GAAGGTCAAGGACAAGATCCAT	GATACGGATGTTTAATCGCTGTC	AGA(6)	141	Yes
PcSSR28	TGGTAGAGAGGGTTTGAGAGTGA	TCAAATGCTCCTTACCGTACCTA	AGA(8)	144	Yes
PcSSR29	CGTTCCTGTTCTAAGGACTTCAA	AAGAAACTCACAGCAACCACAAC	AGAAAG(4)	151	Yes
PcSSR30	AATTTTGAAATTCCGTTCTGTTG	GCAAAAATCCTAATTTTCGTTTTC	AGAAGG(4)	151	Yes
PcSSR31	ATGCGAAAGTGTGTTAGCTTTTC	CCAATCTTTACTACCCACTCCCT	AGG(6)	158	Yes
PcSSR32	GGCAAAATTGGGTAGTGAAATTA	AGTTGTAACTGAGGAAAACCTCG	AGT(9)	148	Yes
PcSSR33	ATCACTATCGTCCAGCCAAGATA	TCAAGAGTAACCAAGGTAGGCAG	AT(10)	135	No
PcSSR34	CAGATGAAACAAAAATTCACACG	GCTCAGATGAAACAAAAATTCACA	AT(11)	145	No
PcSSR35	AACAAACTGTCCCATCAATCAGT	GTTTCCTTGCTAACCAGCATTTA	AT(13)	137	Yes
PcSSR36	TTGCCTGTTAAATGTTGAAGGAC	ATGCAAGAAGAAGCACACCTAGT	AT(8)	145	Yes
PcSSR37	CCATCTAAAACCATTAAGGTCCC	TTACAGCTTCCATCTTTTTCAGC	AT(8)	84	No

PcSSR38	TTCCGAAATTAAGTTGCTTCTCA	AGATTGAACGAAGTGTGACTGGT	AT(8)	145	Yes
PcSSR39	TTATGATTGCATGACATTCTTGG	ATTCATGAGCAATCACCACTTTT	AT(9)	143	Yes
PcSSR40	TAATAGGCCGAGAGGTTGATCTT	AAGAGTTGGGTGAAGTTTTAGGC	AT(9)	118	Yes
PcSSR41	TGGATGACTACAAAGTTTCAGCA	CATGTGTTCCAGGTGATGAGATA	ATAA(5)	131	Yes
PcSSR42	GCCCTAGCTGACTCTACCAAATC	TTTGATAGGGAGGCAAACAAATA	ATC(6)	135	Yes
PcSSR43	AAAGCAATGAAGCAGAGTTCATC	CTGAGAATGATTGGGGTTTTTC	ATC(8)	124	Yes
PcSSR44	TCTACTGAGGTTGGGAATCAGAC	TTGCAACAAATATTACTTCCGCT	ATG(6)	130	Yes
PcSSR45	ACAACAACTTCAAATGTCGATGA	AAGAACAGAGGATATCCCATTGA	ATGGGA(4)	148	Yes
PcSSR46	ATTGAAAACTCAAATTGTCCCAA	ATTGGACTTGATGGTTGCACTAC	ATT(6)	143	Yes
PcSSR47	GACTTTATTGAGCCCCTTCAAAT	GGGATCTATGTCATATTCATCTCTCA	ATTT(5)	139	Yes
PcSSR48	GCCATTTAACCCAAAGGAGTAAG	AGGCTAAGCGGAATTAGGTTTTA	CA(19)	152	Yes
PcSSR49	AAGAACATCTACAAGAGGGGGTC	CTAGCTCTCTGTGGAGGGCCTAT	CA(8)	154	Yes
PcSSR50	AAACCTGAAGGTGTGTTGAAGAA	TGAAGAACAAAAAGACAAAGTTGA	CAAA(5)	143	Yes
PcSSR51	CACCACACATCCTGAATAACCAT	GTACTAGCACTGATCAAGGTGGC	CAC(6)	154	Yes
PcSSR52	TCACCTCCACCAACAACACTAC	CATTGATGATGACCACGTATCAG	CAC(8)	151	Yes
PcSSR53	GGCTCAAACTCAGTCACACCTAC	ATTGTGACATAGCAGAGACAGCA	CAG(7)	158	Yes
PcSSR54	AAAACCACCTAACATTTCACCAA	TATGTGAAGAAGATGACGAGCAA	CAT(6)	118	Yes
PcSSR55	TTCCCCTTCACTTTCTCACATAA	AGCGTCATTTGGAAGAATCAAT	CATTCT(4)	145	Yes
PcSSR56	TTCATCCTCTGAATCACCAGAAC	CAGTCTGTAGAGGGTATGCTTCG	CCA(7)	138	Yes
PcSSR57	GAATGGTAAGAGTAGTGATGGCG	TATCCTGGTAAAACCGATGAACA	CCAGCT(4)	153	Yes
PcSSR58	GTAGTTGCAATTTCCCCTTCTTG	ATACAAAAGAAAGCAATGGCGTA	CCT(6)	155	Yes
PcSSR59	ACCCGTTTACCCTAATACAGCTC	ACTTGTTGCTCTCTTCAGAAACG	CCTTCT(4)	158	Yes
PcSSR60	TTCAAACCCAGATAGCGAACTTA	AAGGAAGTCTCCACCGTATTCTC	CGGTGG(4)	136	Yes
PcSSR61	TAGAAACAGTCCACCTCGAAAAA	CAGCAGGAGTTGGATTTGTAGTT	CT(11)	146	Yes
PcSSR62	ACATTCTCAGTTTCCCAACAGAA	CATAGAAAACACCGTCATCATCA	CT(13)	113	Yes
PcSSR63	CTCACACCAAACAGTCCCTAAAC	GATATTGCGGGAGAGTAGATGG	CT(8)	160	Yes
PcSSR64	TCACTACCTTGGTTCAGTCCATT	CGTTCAACGAGCTGAGTTACAG	CT(9)	138	Yes
PcSSR65	TCAAACCCTCTTCTTCTTCCTTC	TCAGATTCTCTCTTGGGTTTGTT	CTC(7)	145	Yes
PcSSR66	AAAATCCAATGGAGAATACCCAG	CTGTTGAAGGAGATGGTGAAAAG	CTG(6)	113	Yes
PcSSR67	TGCTTCTAGGGCATGTTCTTTAG	AATTCAATATGGAGTTGGAGTGC	CTT(6)	149	Yes
PcSSR68	CTGCAGGTTATTGCATTAGGTTT	GAGTTATTTGAGCATGTGGTTCC	CTT(6)	155	Yes
PcSSR69	AGATCTTCTGTTGTGAACCCTTG	TAAACGAGGCTTCTTTCTTCTCC	CTT(7)	142	Yes
PcSSR70	GCTCTTTATCATCAATCTAGGGC	AACAGGCTTTATTGTTGGTGTTG	CTT(8)	128	Yes
PcSSR71	AGCTCCCCCTAATCTAGCTTTCT	GTTGTGTGTTTTTGTTCCCTCTT	GA(10)	121	Yes
PcSSR72	GCTCTTTCAACTGTGCATTCTTT	TCCAGGTTCTCTCTTTTTATCCC	GA(12)	151	Yes
PcSSR73	AGCTAGCAAATGAGACACCTGAC	TCTCTCACTTTTGCTCTTGCTCT	GA(8)	119	Yes
PcSSR74	ATGTGAAGGGGAGAAAGAAAAAG	ATTCAATTGTAGGCTTGGGAAAT	GA(8)	122	Yes
PcSSR75	AATAAGCAGAGCGGGAATAAAAG	TTTGCTCATGTACTTTCTCTCTTTG	GA(9)	114	Yes
PcSSR76	GCTCAGAACTTTAACCAACCAGA	ACTTTTTCTTTTTCACTTTGGGC	GAA(6)	142	Yes
PcSSR77	AGAAAGAATCAAATCTGGCACTG	CATCTTCTTTCTTGATGTTTCCG	GAA(6)	155	Yes
PcSSR78	TCAAAGTCGTAAAATGAAAGGGA	ATCTTCCAACCCCTTTACACACT	GAA(7)	113	Yes

PcSSR79	AAGACATGATTCATTGCTGGAGT	GATGAACTGAAAACAGCAACACC	GAA(8)	153	Yes
PcSSR80	TGATTGATGTTGTAAGCTATTTGG	TGGCCAGAAAACAAAAAGTAAGA	GAAA(5)	120	Yes
PcSSR81	TGGGAACGTTAAGGAGATACAAA	GGAGATAAGGAAAGGTAGAGCGA	GAAGAT(4)	146	Yes
PcSSR82	GACTTAGAGTTTGCGGTTCAAGA	ACTCTCCTCTTCCACTTCCACTT	GAG(7)	150	Yes
PcSSR83	AAAAGAAAGGAGGGAGGGACTAA	CCCGCTCTCTGATTTTATTCTTC	GAT(7)	141	Yes
PcSSR84	GGGACGGAAATCAGATAACTCTT	TGAGGTTCCACTACTCTTGGAGA	GAT(8)	145	Yes
PcSSR85	AAAAAGAAATCAGCTCCGAAATC	ACCTTTGAATCTAATCACGACGA	GATCT(4)	105	Yes
PcSSR86	CTGAACTAACTTGCACATCCACA	GTGAATGAATCATCAAACAGCAA	GCA(8)	100	Yes
PcSSR87	CATCCTGTAAATTTATGCTGCTTG	TGAATATAGCCATTTTCTTTTAGC	GCTAA(4)	160	Yes
PcSSR88	GGAAAGGAATATAAGGGAAAGGA	AGAAGGTGACTGGAATCTTCACC	GGAAAG(5)	152	Yes
PcSSR89	GTGGGATTGAACCTGACTTTGTA	GTCCTCCCAATTCTTCTACTGCT	GGAGGT(4)	150	Yes
PcSSR90	GAGGAGCTAAAAGAAGAAGGGTG	CTTCAAGTTAATAGAGCCCCTGC	GGGAAG(4)	156	Yes
PcSSR91	TTGATTGATTCACAGGAAAAGGT	GAGGAGAGTCATGTGGTATGTCC	GGT(6)	144	Yes
PcSSR92	ATTGTTTGAATAAGGTGGAGGTG	GTAAGGTGGAATCTTTAAAGCCG	GGTGGA(4)	155	Yes
PcSSR93	TTCTTGAAGGAGCTCCAGAAGTT	CTCCACAACATCCTCCTCATC	GTG(6)	131	Yes
PcSSR94	TTCATCTGACAGAAAACCCAAAT	TTCATCCCAGTCTATACCCGATA	GTG(6)	158	Yes
PcSSR95	TCAAAATTCAGGTTCACTCCACT	CAAACACCTTCATCACACTACCA	GTT(7)	149	Yes
PcSSR96	TTTTCATGGATTGATGTGAACTG	ACCGTCAATCACAATCTTGTTTC	GTTTA(4)	156	Yes
PcSSR97	GATGCCATGAACTTCCACTCTTA	TGTTACATGCGAGAGAAACTGAA	T(31)	157	Yes
PcSSR98	TGTAACTTGTCTCAAGGGTGCTT	CATAGTGGTCATACTGGGTCACA	TA(10)	121	Yes
PcSSR99	TGCTGATTCTTTACTGTCCATGA	AACATTTCTTGTGACAAATGGACT	TA(11)	148	Yes
PcSSR100	TCACAAAAGAAAAAGAAAAACGC	TCACAAAAGAAAAAGAAAAACGC	TA(12)	148	No
PcSSR101	GAATACGAGATTGGCATCACACT	CCTTGAGTCAAAAGGCTGTAAGA	TA(8)	142	No
PcSSR102	AAACAGACCCCATTTCATTTCTT	CTTGGTCATGTAGGGTTAGCAAG	TA(8)	152	Yes
PcSSR103	GTAAAATCCATAACCATTTGCCA	AACATTTGTCAGAAGCATTTTCC	TA(8)	157	Yes
PcSSR104	CACAAATAAACCAGCTTTACGGA	CACTCTGCCGTTGATAAAGTTTC	TA(9)	133	No
PcSSR105	CAGATCACATTACCCAGATCACA	TAGTGTAATCAATGAACTGGGGC	TA(9)	142	No
PcSSR106	TTCCATTGATGTACCCATGAACT	TTTTTGACATCACAACTGACTCG	TAA(6)	160	Yes
PcSSR107	TGAAAAGAGGAATAAGAGTAATGCAA	TGAAGACAAGAAGAGGAGAGGTG	TAAA(5)	154	No
PcSSR108	CTCGGTTTCTTCTCTGGATTTTT	GGAGCTTCGTAAAACAAGGATTT	TAG(7)	126	No
PcSSR109	GGCTTTATCCATTTGTCTCCATT	TTGCTTGTTTACGTGTTCATTTG	TAT(6)	111	Yes
PcSSR110	CTCTTACATCCATCATCCTCCTG	GAAATGGAGATGGCATTTGATTA	TATT(5)	145	Yes
PcSSR111	GACTTCGAATTAGAGCCAATCAA	GATTAGATGGCAAACACAGAGAGA	TC(10)	94	Yes
PcSSR112	TCCAATCCCAGTCTTCTAGACCT	ACTGATGCCTAAAGGAGCACATA	TC(10)	157	Yes
PcSSR113	TTCCCCCTATAACATTTCAAACA	AACATCAAACGAAAGAGCAGAAA	TC(12)	120	Yes
PcSSR114	GCTCCTTTCTCATTTATGGGATT	CTCAGAAGCGATTCCTACGTCTA	TC(17)	140	Yes
PcSSR115	ACCATGAGAACGAAAACCCTAAT	CCATATTATCCAACCAGAAAATGA	TC(8)	145	Yes
PcSSR116	CAAAGAAGCCAACAAAGAAGGTA	AAACAAAAGAAAAACGGAAAAAGA	TC(8)	102	Yes
PcSSR117	TCAATTCAAATTGGTCTCTGCTT	TATGGAGGTATGGTATGGTGTCC	TC(9)	147	No

PcSSR118	TATTGGTTTCAAAATCACAAGGC	CGTCTTTCCTGTCAATGATTTCT	TCA(7)	142	Yes
PcSSR119	TCTCAACAGAAACACCATGTCAG	AGAAGACTTTTGGGTTTGGTAGC	TCACAA(4)	150	Yes
PcSSR120	CAACATCAAGCTCATTCTCCTCT	GCTGTTCTTGATCTTCATTTGCT	TCC(6)	158	Yes
PcSSR121	GACCATCATAAGAACCCTGAACA	CGGATTGGATTCTGAGAGTAATG	TCCCTA(4)	152	Yes
PcSSR122	TCCTCATCTGAAGCTCCTAACAC	CACAAAACCACACATCATAATCG	TCT(6)	156	Yes
PcSSR123	GCATTTGCAAACCATTAAAAGAT	AGGTGAATGCTGAAGACAGAGAA	TCT(6)	123	Yes
PcSSR124	ATCAAATACCCTTTCAAGCCTTC	ACTGGATCAGGTGATGGAGTAGA	TCT(7)	113	Yes
PcSSR125	TTGCGCAAACTAATTAAAAGCTC	TCTGAGAGGTAGATTCTGCATGG	TCT(8)	122	Yes
PcSSR126	ACCCCCAATTAAAATCAGATCAC	ATGGAATGTTTGATTTTGCCTAA	TCTGAC(4)	159	Yes
PcSSR127	AATTCAATGATGTTGAAAGGGAA	TGATATCACTAAATCAAACCCAAAA	TG(10)	106	Yes
PcSSR128	AAAGCTAAATCCTTCAATGCCTC	AATTTCAAATGGGCAAATTCTTT	TG(8)	158	Yes
PcSSR129	CCGTAATCCGAGAAGGTAAAAAT	CCCATCTACATGTTCAACACAGA	TG(9)	150	Yes
PcSSR130	GGATCTGCGTTCAAGAAACATAC	CATGGCAGACTGTACTCTTGATG	TGA(7)	151	Yes
PcSSR131	CAACACAATCTTCCCTTCATTTC	AGAGTGTTTGTGTTTGTGCTTCA	TGA(9)	160	Yes
PcSSR132	TTCTCCTCCTTCTGCTTCTTCTT	TCTCAGTTCTCTGAAAATGGCTC	TGC(6)	132	Yes
PcSSR133	AGCAGGGAGATGGTAGGAGTAGT	CTAACAACAGCGCCAATCTTATC	TGG(6)	160	Yes
PcSSR134	TTGATTCCATTTCTCCACTTTGT	AAAGGTCGAATTCCTGATTGATT	TGG(7)	148	Yes
PcSSR135	CTTCTTGCATCTAGCACCAAGTT	TCAAAAATAGAAACAGACGACAGG	TGGATC(4)	100	Yes
PcSSR136	TTGTTTTCTCCGAACTGGTTAAA	AAACAACAAATAAAACAAAGAAGCG	TGT(7)	137	Yes
PcSSR137	GTTTTTCTTTTCCTTCTTGATGC	TAAGGGTTCGTTCATTTGTGTTT	TTA(6)	157	Yes
PcSSR138	ATGGACATTCAAATCGCATAGAC	ATCGTTGCTCTACGATCTCTACG	TTC(11)	120	No
PcSSR139	TCCAGAGAAAAATGGGATGATTA	AACGCAAGTCAAAGTGCTAAAAG	TTC(6)	138	Yes
PcSSR140	AGTCTACCATTCAATTCCCGAGT	AATCCTCACACTGCAGCAGATAG	TTC(6)	141	Yes
PcSSR141	AACCAACTAAATGCACTCCAAAA	TATCGTCTTCCAGCTCAAAACTC	TTC(6)	114	Yes
PcSSR142	AATTTGACTCCACCAAGTTTGAA	AGGAGCAAAATTAAGGAGGAGAA	TTC(6)	158	Yes
PcSSR143	CCACCTGCCATCTGTAACTTTT	CTTCACAAGAGGTTGAACGTAGG	TTC(8)	159	Yes
PcSSR144	ATCACACAAGAGGGAGTGAATGT	TCCAATGGCTTAATAGGAACAAA	TTCT(5)	136	Yes
PcSSR145	CAGTGGAACTGCTTTCAGAGTTT	GAGAACTACAATGTTTTGCGGAC	TTG(6)	160	Yes
PcSSR146	CTCTAGAGTTCGGAACAGCGTC	GATTCCATGAGGAAGAGTGTGAG	TTG(7)	160	Yes
PcSSR147	TGAAGACAAGAAGAGGAGAGGTG	TGAAAAGAGGAATAAGAGTAATGCAA	TTTA(5)	154	No
PcSSR148	TGACCTCGTTATGACAAAATTCA	TGAAGTAGTTTCTTATTCGCCCC	TTTA(6)	148	Yes
PcSSR149	AGGCTTAACCCACTTCTTGTCTT	ACCTTGTTTGAGCAGGTTTTACA	TTTC(5)	160	Yes
PcSSR150	AACACTCCAACCGCAGATATTTA	CCAGCAACTTTGCATAGAGATTA	TTTCTC(4)	158	Yes
PcSSR151	GGGTTTTGCATGTTAATCATTTC	CCCTTTCTCTCTTCAGAACCCTA	TTTTTC(4)	136	Yes
